# Coronary endothelial function is directly related to extent of weight loss in obese patients

**DOI:** 10.1186/1532-429X-15-S1-O44

**Published:** 2013-01-30

**Authors:** Allison Hays, Sahar Soleimanifard, Michael Schär, Gary Gerstenblith, Kerry Stewart, Matthias Stuber, Robert G Weiss

**Affiliations:** 1 Medicine, Johns Hopkins, Baltimore, MD, USA; 2 Radiology, University of Lausanne, Lausanne, Switzerland; 3 Radiology, Johns Hopkins, Baltimore, MD, USA; 4 Philips Healthcare, Cleveland, OH, USA

## Background

Obesity is a risk factor for future cardiovascular events.[1] Prior studies have shown that weight loss is related to improved peripheral vascular function.[2] However, the relationship of weight loss to coronary endothelial function, which is impaired in early atherosclerosis and predicts future cardiovascular events,[3] is not completely understood. By means of previously described non-invasive MRI methods combined with isometric handgrip to assess endothelial-dependent coronary vasoreactivity,[4]4 we tested the hypothesis that weight loss over six months is associated with better coronary endothelial function (EndoFx) in obese, non-diabetic individuals.

## Methods

**Figure 1:**
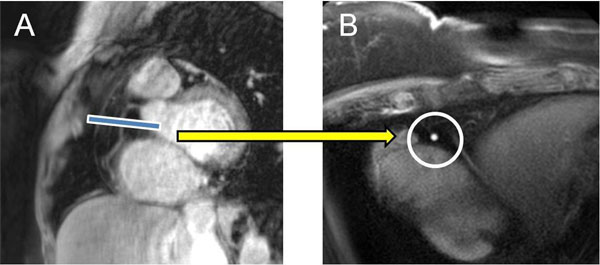
Typical anatomical bright blood coronary images using MRI. In an obese subject with body mass indea of 35, in image **A**, a scout scan obtained parallel to the RCA (right coronary artery) is shown with the location for cross-sectional imaging (blue line). In **B**, a corresponding coronary cross section is shown (circled in white) from which area measurements are made before and during isometric handgrip stress.

Eighteen overweight and obese non-diabetic adults defined as BMI >25 (BMI=32.3±4.4, age=54.2±8.1 years, mean±SD) with no history of cardiovascular disease underwent a six month diet and exercise weight loss regimen. At the end of the study period, participants were imaged using a 3T MRI scanner (Achieva,Philips,Best,NL) and a 32-element cardiac coil for signal reception(Figure 1). To measure EndoFx, spiral coronary MRI was performed before and during continuous isometric handgrip exercise, an endothelial-dependent stressor.[5] Coronary cross-sectional area (CSA) and coronary blood velocity (CV) changes were measured, and coronary blood flow (CBF) was calculated as previously reported.[4] A cholesterol panel and weight data were obtained at the beginning and end of the study period.

## Results

**Figure 2:**
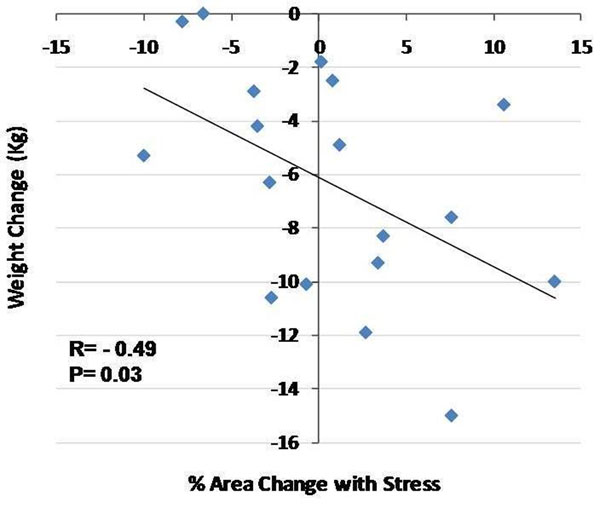
% coronary artery area change with isometric handgrip stress is directly related to extent of weight change over six months in overweight adults (N=18).

Sixteen of 18 patients completed full adherence to the six month weight loss regimen whereas 2 dropped out before intervention ended. Nevertheless, MRI and weight loss data were obtained in all 18 participants at the time of their study termination. The average weight loss for all subjects was 7.1±4.1 kg (range=0-15 kg). We detected a direct relationship between the degree of weight change and % CSA change with stress (r=-0.49, p=0.03, Figure 2). There was also a direct association between weight change and % change CBF (r=-0.46, p=0.05). There was no significant association between triglyceride change and % CSA change (r=-0.17, p=0.25).

## Conclusions

Using 3T MRI combined with isometric handgrip exercise to quantify coronary endothelial-dependent vasoreactivity, we observed a significant relationship between the degree of weight change and coronary endothelial function as quantified by % CSA and % CBF change during handgrip stress. The present findings demonstrate that weight loss in obese and overweight subjects is correlated with better coronary EndoFx, independent of changes in triglyceride level. This MR approach permits the noninvasive study of EndoFx in obese patients and may offer important insights into the effects of long term therapeutic or lifestyle obesity interventions on coronary vascular function and atherosclerosis.

## Funding

NIH/NHLBI (ROIHL084186, ARRA 3R01Hl084186-04S1) and American Heart Association.
